# Patient selection guidelines for magnetic resonance focused ultrasound (MRgFUS) treatment: an updated view

**DOI:** 10.1186/2050-5736-3-S1-P86

**Published:** 2015-06-30

**Authors:** Kelli Bryant, Suzanne LeBlang

**Affiliations:** 1 University MRI, Boca Raton, Florida, United States

## Background/introduction

The purpose of this retrospective study was to evaluate the selection criteria used to determine patient eligibility for MRgFUS for the treatment of symptomatic uterine fibroids.

## Methods

373 women with symptomatic uterine fibroids were screened with MRI exams with and without contrast utilizing T2 coronal and axial, T2 fat suppressed sagittal, T1 axial precontrast images, and post contrast fat saturated images in 3 planes. Patients were initially considered clinically eligible if they met the standard published screening criteria/Food and Drug Administration-based treatment guidelines. These selection criteria were expanded on a case-by-case basis if it was felt that a NPV of at least 50% could be obtained or if the patient refused other treatment approaches (myomectomy, hysterectomy, uterine artery embolization).

## Results and conclusions

Results: Of the 373 patients (ages 26-61) that underwent pelvic screening, 188 (51%) were considered eligible and 135 (36%) were excluded for the MRgFUS procedure based on the published selection criteria. An additional 50 patients (13%) would have been excluded from treatment based on the published criteria, but were given the opportunity for MRgFUS treatment. Of those 50 patients, 35 patients were treated, with 33 having fibroids >10 cm, 1 having prior liposuction and 1 having multiple small fibroids. Of the 33 patients with fibroids in excess of 10 cm, 11 agreed to have pre treatment with a GnRH agonist while the other 22 patients refused all other interventions besides MRgFUS. 135 patients (36%) were excluded from treatment. The reasons for exclusion are listed in Table 1 below. Using the FDA-based treatment guidelines, 188 fibroids were treated with MRgFUS with an average NPV/fibroid of 66%. Using expanded selection criteria, an additional 60 fibroids were treated with an average NPV/fibroid of 56%.

**Table 1:** Patients excluded from MRgFUS treatment following MRI screening

Reason for Exclusion	Percentage of Patients (%)	Number of Patients
Fibroid number (>6)	33	45

Possible malignancy	14	19

Diffuse adenomyosis	12	16

Non-enhancing fibroids (calcified, hemmorrhagic, necrotic)	11	15

Fibroid size > 10 cm	9	12

Fibroid size < 2 cm	6	8

Pedunculated fibroid	4	5

Hyperintense T2-weighted signal	5	7

Metal in beampath	3	4

Fibroid location > 12cm from skin	1	2

Obesity {subcutaneous fat layer > 4.0 cm)	1	1

Prior Liposuction	1	1

Conclusions: Using the published selection criteria, 51% of patients who underwent MRI screening were considered candidates for MRgFUS. An additional 13% of patients were brought into the treatment arm of the study after pre-treatment with a GnRH agonist or after considering patient preference. This individualized approach to determining patient eligibility resulted in only a slightly smaller average NPV/fibroid of 56% *vs*. 66% under the published selection criteria. As physicians acquire more clinical experience with MRgFUS, an expanded selection criterion may allow more women to benefit from this more conservative approach to treating uterine fibroids.

